# Correction: Optical, dielectric & ferroelectric studies on amino acids doped TGS single crystals

**DOI:** 10.1039/d5ra90058a

**Published:** 2025-05-15

**Authors:** P. R. Deepthi, J. Shanthi

**Affiliations:** a Department of Physics, School of Engineering, Presidency University Bengaluru Karnataka 560 089 India deeptiprasad82@gmail.com; b Department of Physics, Avinashilingam University for Women Coimbatore Tamilnadu 641 038 India

## Abstract

Correction for ‘Optical, dielectric & ferroelectric studies on amino acids doped TGS single crystals’ by P. R. Deepthi *et al.*, *RSC Adv.*, 2016, **6**, 33686–33694, https://doi.org/10.1039/C5RA25700J.

The authors regret that Fig. 1a and c were reproduced from ref. 1 below without being correctly attributed in the figure caption. Ref. 1 was not cited in the original article. The correct figure caption is shown below:

**Fig. 1** The photograph of the grown crystals of (a) pure TGS, (b) L-Ar TGS, (c) L-H TGS and (d) L-Al TGS. Images (a) and (c) reproduced *via* a Creative Commons Attribution 4.0 International License [ref. 1].

The Royal Society of Chemistry apologises for these errors and any consequent inconvenience to authors and readers.
